# Deoxybouvardin Glucoside Induces Apoptosis in Oxaliplatin-Sensitive and -Resistant Colorectal Cancer Cells via Reactive Oxygen Species-Mediated Activation of JNK and p38 MAPK

**DOI:** 10.4014/jmb.2410.10008

**Published:** 2025-01-30

**Authors:** Seung-On Lee, Sang Hoon Joo, Jisu Park, Quan T. Khong, Si Yeong Seo, Goo Yoon, Jin Woo Park, MinKyun Na, Jung-Hyun Shim

**Affiliations:** 1 Department of Biomedicine, Health & Life Convergence Sciences, BK21 Four, College of Pharmacy, Mokpo National University, Muan 58554, Republic of Korea; 2 College of Pharmacy, Daegu Catholic University, Gyeongsan 38430, Republic of Korea; 3 College of Pharmacy, Chungnam National University, Daejeon 34134, Republic of Korea; 4 Department of Pharmacy, College of Pharmacy, Mokpo National University, Muan 58554, Republic of Korea; 5 The China-US (Henan) Hormel Cancer Institute, Zhengzhou, Henan 450008, P.R. China

**Keywords:** Deoxybouvardin glucoside, reactive oxygen species, colorectal cancer, JNK, p38 MAPK, apoptosis

## Abstract

The roots of *Rubia* spp. (Rubiaceae) have been employed to treat hematemesis, inflammatory disease, and tumor. Cyclohexapeptides derived from *Rubia* spp. have been reported to have antitumor potential; however, the mechanism of action for their antitumor activity remains unclear. We aimed to examine the antitumor effect of deoxybouvardin glucoside (DBG), a cyclohexapeptide from *Rubia* spp. on oxaliplatin (Ox)-resistant human HCT116 colorectal cancer (CRC) cells. Cell viability in the presence of DBG was monitored using an MTT viability assay, and flow cytometry was used to analyze changes in apoptosis, cell cycle, mitochondrial membrane potential (MMP), and reactive oxygen species (ROS) activity. The antiproliferative activity involved apoptosis and phosphorylation of JNK and p38 MAPK. Inhibition of JNK and p38 MAPK by specific inhibitors prevented DBG-induced apoptosis, underscoring the close involvement of these kinases. Further, DBG induced cell cycle arrest in CRC cells at the G2/M phase by regulating the p21, p27, cyclin B1, and cdc2 proteins. DBG-induced apoptosis was accompanied mitochondrial membrane depolarization, resulting in cytochrome c release into the cytoplasm and caspase activation. Remarkably, DBG induced apoptosis by generating high ROS levels. The mediation of apoptosis by increased ROS generation was confirmed by pretreatment with the ROS scavenger N-acetyl cysteine (NAC). Collectively, DBG exhibited anticancer activity against both Ox-sensitive and Ox-resistant CRC cells by targeting JNK and p38 MAPK, inducing cell cycle arrest, elevating cellular ROS levels, and disrupting MMP. This study suggests that DBG has the potential to be utilized as a therapeutic agent for treating Ox-resistant CRC.

## Introduction

**Fig. 1:**
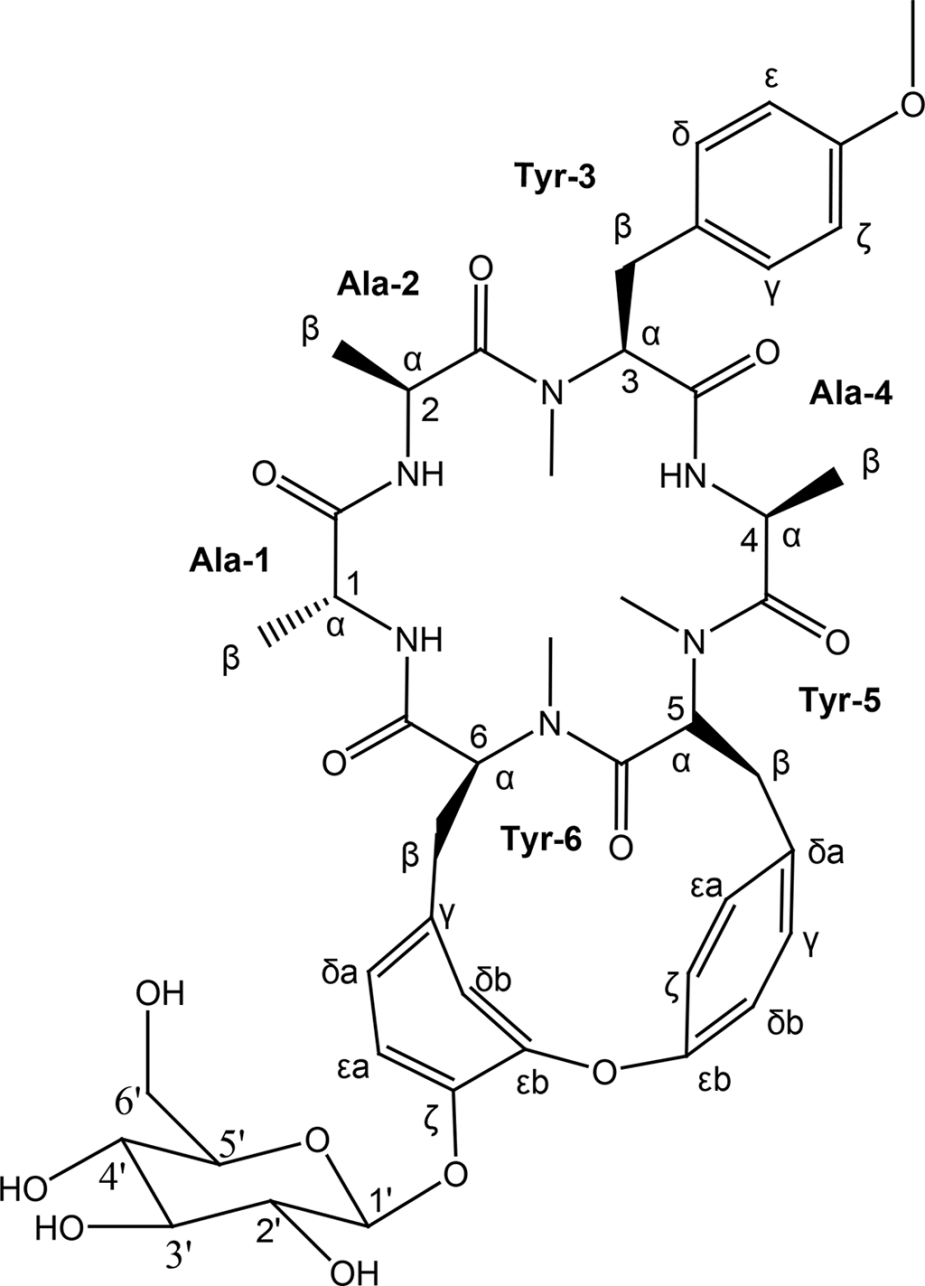
Chemical structure of deoxybouvardin glucoside isolated from *R. philippinensis*.

Numerous natural products have been shown to regulate various cell signaling pathways and have been used as adjuvants for preventing or curing chronic diseases or enhancing anticancer activity [1]. Rubiae Radix, the roots of *Rubia akane* Nakai (= *R. cordifolia* L. var. *mangista* Miq.) (Rubiaceae) is a traditional medicine listed in Korean Herbal Pharmacopoeia, which has been utilized to treat hematemesis, various inflammatory diseases, and tumor. In Chinese traditional medicine, the roots of *R. cordifolia* L. have been used for their antiproliferative and anti-inflammatory activity in treating rheumatism, inflammation, psoriasis, and other conditions [2], and many biologically active compounds could be obtained from the genus *Rubia* [3]. Hundreds of chemical constituents such as anthraquinones, naphthoquinones, terpenoids, and cyclopeptides have been reported from the genus Rubia, some of which were responsible for the pharmacological effects of *Rubia* spp., in particular, antitumor, anti-inflammatory, anti-osteoporotic, anti-adipogenic, and anti-platelet aggregation activities [2]. Deoxybouvardin glucoside (DBG, Fig. 1) is a cyclic hexapeptide glycoside originally isolated from *Bouvardia ternifolia* [4]. Since then, several other deoxybouvardin derivatives with antitumor activity have been found in the genus *Rubia* [5, 6]. Early studies indicate that deoxybouvardin (DB) has anticancer [7] and anti-inflammatory activities [8]. Further, DB exerts anticancer activity in human breast cancer cells by inducing apoptosis via a PI3K/AKT pathway-dependent mitochondrial pathway [4]. Recently, we investigated the antiproliferative activity of DB in colorectal cancer (CRC) cells [9]. We found it intriguing to compare the anticancer activity of DB with that of its glycoside derivative, DBG. It was expected that the addition of glucose moiety to DB may alter the behavior of the molecule, especially the cellular uptake of DBG if necessary.

CRC is prevalent worldwide, accounting for approximately 577,000 deaths in 2020 [10]. Two million new diagnoses of CRC are estimated by 2035. Chemotherapy is a key method for treating patients with CRC who are not eligible for surgical treatment, and can improve overall survival by controlling cancer progression [11]. Currently, several treatment regimens are available, including monotherapy with 5-fluorouracil and combination therapy with oxaliplatin (Ox), irinotecan, and capecitabine [12]. However, chemotherapy has drawbacks, such as systemic toxicity, unpredictable resistance, and inadequate cancer specificity. Ox is a third-generation, platinum-based antineoplastic agent that ameliorates the drawbacks of cisplatin and carboplatin [13]. In Ox, the two ammine ligands are replaced by trans-1,2-diaminocyclohexane, which imparts different cancer specificities and non-targeted cytotoxicity [14]. The mechanism of Ox involves crosslinking of RNA, protein, and DNA molecules. In particular, formation of an intra-strand adduct between two neighboring purine bases (G-G, or G-A) inhibits the replication or transcription of DNA. The larger structure of Ox compared to that of cisplatin results in the formation of fewer DNA adducts. However, Ox has higher toxicity, and improvement in cancer specificity is set off by its increased toxicity. Moreover, intrinsic and acquired resistance also exist in Ox-based combination therapy, which hamper treatment efficacy [15]. Therefore, urgent attention is needed for developing more effective and less toxic anticancer therapeutics that also address Ox resistance.

In this study, we challenged the Ox-resistant human CRC HCT116 cells (HCT116-OxR) to determine the mechanisms by which DBG induces cell death. DBG induced apoptosis by elevating reactive oxygen species (ROS) levels, thereby activating the JNK and p38 MAPK signaling pathways. These results underpin the development of DBG as a potential therapeutic agent against Ox-resistant CRC.

## Materials and Methods

### Chemical

Deoxybouvardin glucoside (DBG; purity of 95%, Fig. 1) was isolated both from the roots of *R. akane* and *R. philippinensis* as previously described [16]. The features and chemical structure of DBG were determined using MS and NMR spectroscopy (Table 1): yellowish amorphous powder; ^1^H and ^13^C NMR data (CD3OD, 300 and 75 MHz); ESIMS, *m/z* 919.3 [M + H]^+^, *m/z* 941.3 [M + Na]^+^, *m/z* 917.3 [M - H]^-^; see Supporting Information (Figs. S1–S3).

### Reagents

Dulbecco's modified Eagle’s medium (DMEM), and minimum essential medium (MEM), and RPMI-1640 were purchased from Welgene (Republic of Korea). Trypsin, penicillin/streptomycin (p/s), MEM vitamin solution, sodium pyruvate, fetal bovine serum (FBS), and MEM nonessential amino acid solution were obtained from Hyclone (USA). Phosphate-buffered saline (PBS), RIPA buffer, and Tris-glycine-SDS buffer were purchased from Bio-Solution (Republic of Korea). Dimethyl sulfoxide (DMSO), N-acetyl cysteine (NAC), 1-(4,5-dimethylthiazol-2-yl)-3,5-diphenylformazan (MTT), SP600125 (JNK inhibitor), Z-VAD-FMK, a pan caspase inhibitor, and SB203580 were obtained from Sigma-Aldrich. Antibodies against β-actin, 78-KDa glucose-regulated protein (GRP78), C/EBP homologous protein (CHOP), p21, p27, cyclin B1, cdc2, Mcl-1, Bid, Bax, Bad, Bcl-xL, Bcl-2, cytochrome c (cyto c), β-tubulin, cytochrome c oxidase subunit 4 (COX4), Apaf-1, poly (ADP-ribose) polymerase (PARP), and caspase3 were purchased from Santa Cruz Biotechnology (USA). Phospho (p)-JNK, JNK, p-p38, and p38 antibodies were provided by Cell Signaling Technology (USA).

### Cell Culture and Treatment

HCT116 (CCL-247) and human primary epidermal keratinocyte (HEKa) (PCS-200-011) cell lines were procured from the American Type Culture Collection (USA). The human oxaliplatin-resistant colorectal cancer cell line (HCT116-OxR) was provided by the University of Texas, MD Anderson Cancer Center [17]. HCT116 cells were maintained in RPMI-1640 containing 100 U/ml p/s and 10% FBS. HEKa cells were cultured in DMEM supplemented with 100 U/ml p/s and 10% FBS. HCT116-OxR cells were cultured in MEM containing FBS (10%), MEM nonessential amino acid solution (1%), sodium pyruvate (1%), MEM vitamin solution (1%), and p/s (1%). All cell lines were cultured at 37°C with 5% CO_2_. DBG was dissolved in DMSO to prepare stock samples and diluted to the final concentrations for each experiment. CRC cells were cultured at a density of 5,000 (HCT116) or 4,000 cells per well (HCT116-OxR) in six-well plates and incubated overnight before treatment. Unless otherwise indicated, cells were harvested for analysis after exposure to DBG for 48 h.

### MTT Assay

MTT assays were used to measure cell viability after exposure to varying concentrations of DBG (0, 3, 6, and 12 nM). HCT116 (5 × 10^3^ cells/well), HCT116-OxR (4 × 10^3^ cells/well), and HEKa (8 × 10^3^ cells/well) cells were seeded into 96-well plates for 24 h, followed by DBG treatment (24 or 48 h). When the effect of inhibitors was tested, pretreatment with or without inhibitors (NAC, SP600125, SB203580, or Z-VAD-FMK) for 3 h was followed by DBG (12 nM) treatment. After adding MTT reagent to each well, the plates were maintained at 37°C for 40–90 min. Finally, the resulting formazan was dissolved in DMSO and the plates were spectrophotometrically analyzed using a MULTISKAN GO system (Thermo Fisher Scientific, Finland) at an absorbance of 570 nm.

### Soft Agar Assay

Cells (8,000 cells/well) were cultivated with various DBG concentrations (3, 6, and 12 nM). The plates were coated with 0.6% agar as a bottom layer containing DBG or Ox (2 μM). Cells were suspended in 0.3% agar containing DBG or Ox and seeded on the agar layer. The cells were then maintained for 10 d to form cell colonies. Photographs of the colonies were captured using a software LAS V4.10 (Leica Microsystems, Germany). Colony size and number were determined using i-Solution software (IMT i-Solution, Canada).

### Western Blotting

Cells were extracted in RIPA buffer containing protease and phosphatase inhibitors. The protein concentration of each sample was determined using Bio-Rad BCA protein assay kit (USA). Cellular proteins were then resolved by SDS-PAGE (Bio-Rad) and transferred to Millipore PVDF membranes (USA). Bands were visualized with an Image Quant system (LAS500; GE Healthcare, Sweden). Proteins were quantified using ImageJ (National Institutes of Health, USA).

### Cell Cycle Analysis

Propidium iodide (PI) staining assays were used to investigate cell cycle phase distribution. Cells were collected and fixed with 70% ethyl alcohol at -20°C overnight. Cells were then washed and resuspended in PBS with RNase A for 30 min at 37°C. After staining with a cell cycle reagent (Muse Cell Cycle Kit, MCH100106, Luminex, USA) for 30 min, cell cycle phase distribution was assessed using a Muse cell analyzer (Merck Millipore, Germany).

### Reactive Oxygen Species (ROS) Measurement

Intracellular ROS levels were determined using a Muse Oxidative Stress Kit (Luminex, MCH100111). The treated cells were incubated with the Muse Oxidative Stress Reagent working solution for 30 min at 37°C in the dark. ROS levels were then assessed using a Muse cell analyzer.

### Mitochondrial Membrane Potential (MMP) Assay

MMP changes were measured by staining with JC-1 dye from Thermo Fisher Scientific (USA). Cells were exposed to various concentrations of DBG for 48 h in a six-well plate, collected, and resuspended with JC-1 working solution containing JC-1 at 2 μg/ml for 15–30 min incubation at 37°C. The cells were then washed with PBS and analyzed by flow cytometry (MACSQuant Analyzer; Miltenyi Biotec, Germany).

### Isolation of Cytosolic and Mitochondrial Fractions

The cells were harvested and suspended in plasma membrane extraction buffer [10 mM HEPES (pH 8.0), 250 mM sucrose, 10 mM KCl, 1.5 mM MgCl_2_∙6H2O, 1 mM EGTA, 1 mM EDTA, 0.1 mM phenylmethylsulfonyl fluoride, and 0.01 mg/ml each of leupeptin and aprotinin]. The suspended cells were disrupted by adding digitonin (0.1%) followed by incubation for 5 min on ice. The cytosolic fraction was centrifuged at 13,000 rpm for 5 min. The pellet containing mitochondria was resuspended in plasma membrane extraction buffer containing 0.5% Triton-X 100 and centrifuged at 13,000 rpm for 30 min at 4°C.

### Annexin V/7-Aminoactinomycin D (7-AAD) Staining

Annexin V staining was performed using Muse Annexin V & Dead Cell Reagent (Luminex, MCH100105). The cells were harvested after trypsinization, washed with 1X PBS, and then stained with Muse Annexin V and Dead Cell Reagent for 20 min in the dark. Apoptotic cells were determined using a Muse cell analyzer.

### Multi-Caspase Assay

The activity of caspases was detected using a Muse MultiCaspase Kit (Luminex, MCH100109). The cells were stained with the Muse MultiCaspase Reagent for 30 min. After incubation, Muse Caspase 7-AAD working solution was applied, and caspase activity was analyzed using the Muse cell analyzer.

### Statistical Analysis

All data are presented as the mean ± standard deviation (SD) from triplicate experiments, and data were analyzed by one-way or two-way analysis of variance (ANOVA) with Tukey’s multiple comparison test. A *p*-value of *p < 0.05, **p < 0.01, and ***p < 0.001 indicated a statistically significant difference compared with the control groups. Significant differences between the drug and inhibitor treatments were denoted as ^#^*p* < 0.05, ^##^*p* < 0.01, and ^###^*p* < 0.001.

## Results

### DBG Inhibits the Survival and Colony Formation of CRC Cells

**Fig. 2:**
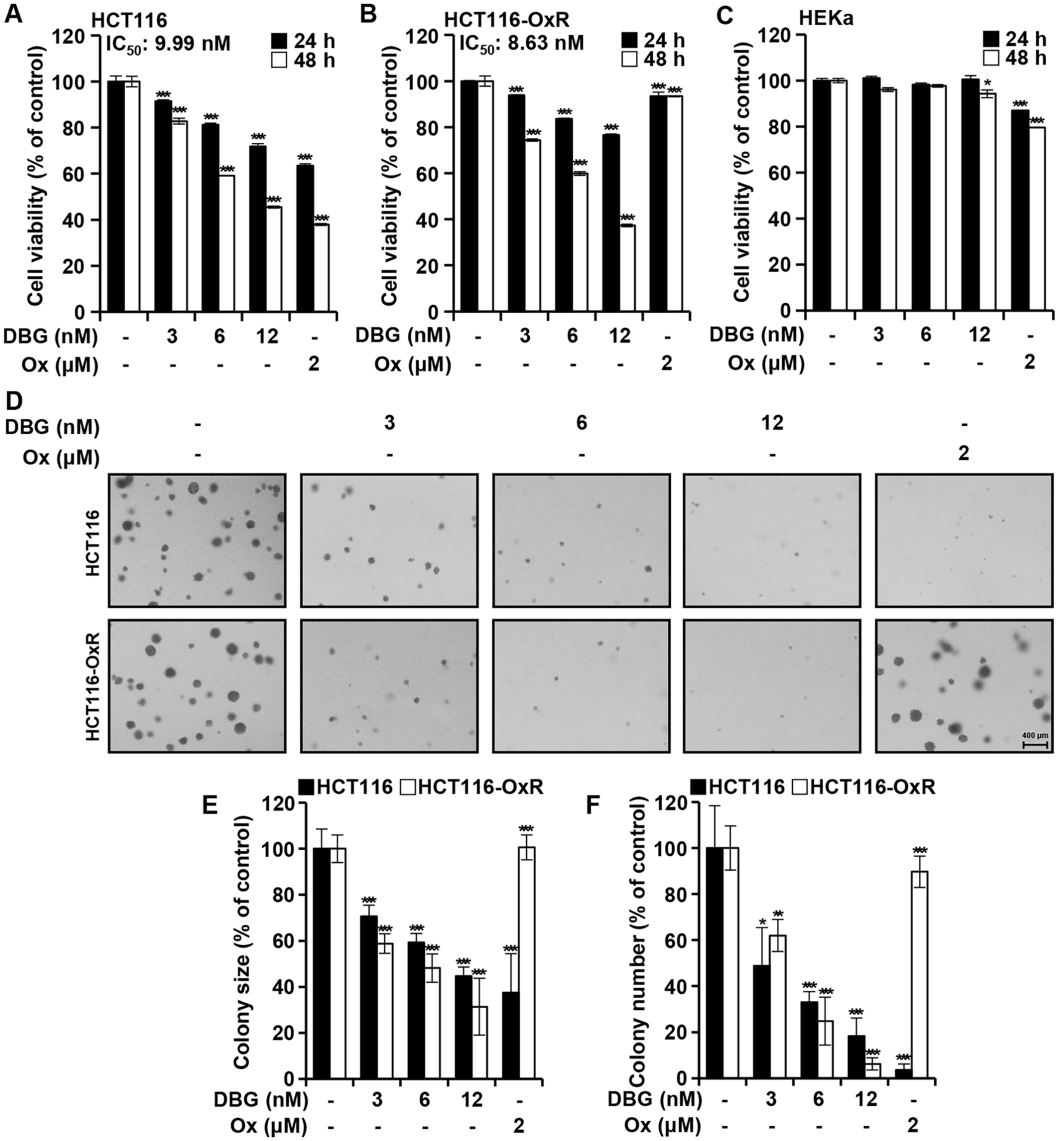
Cell viability of colorectal cancer (CRC) cells in the presence of deoxybouvardin glucoside (DBG). (**A-C**) Cell viability of HCT116/HCT116-OxR and HEKa cells treated for 24 (solid) and 48 h (empty) with DBG (0, 3, 6, and 12 nM) and Ox (2 μM) as indicated, based on the MTT cell viability assay. Data shown as the mean ± SD (*n* = 3). *p < 0.05 and ***p < 0.001 compared to the control. (**D–F**) Anchorage-independent colony growth was determined using a soft agar assay (10 days incubation). (**D**) Micrographs. (**E-F**) Size and number of colonies. *p < 0.05, **p < 0.01, and ***p < 0.001 compared to the control.

The viability of HCT116 and HCT116-OxR cells treated with DBG (0, 3, 6, and 12 nM) for 24 and 48 h was measured. In HCT116 cells, viability decreased by 82.73%, 59.07%, and 45.45% with 3, 6, and 12 nM DBG, respectively, compared to the control and showed an IC_50_ of 9.99 nM. In HCT116-OxR cells, the corresponding values were 74.33%, 59.93%, and 37.30%, respectively, with an IC_50_ of 8.63 nM. As shown in Fig. 2A and 2B, the survival rate was higher in HCT116-OxR cells than in HCT116 cells in the presence of Ox (2 μM). Further, DBG did not significantly alter the viability of HEKa cells (Fig. 2C).

A soft agar assay was used to determine anchorage-independent survival. Micrographs showed that DBG treatment decreased the anchorage-independent survival of CRC cells in a dose-dependent manner (Fig. 2D), along with colony size and number (Fig. 2E and 2F). In the Ox-treated group, colony formation was lower in HCT116 cells than in HCT116-OxR cells, both in number and size. These results indicate that DBG inhibited the survival and colony formation of Ox-resistant as well as Ox-sensitive CRC cells.

### DBG Induces Apoptosis in CRC Cells through JNK/p38 MAPK Activation

**Fig. 3:**
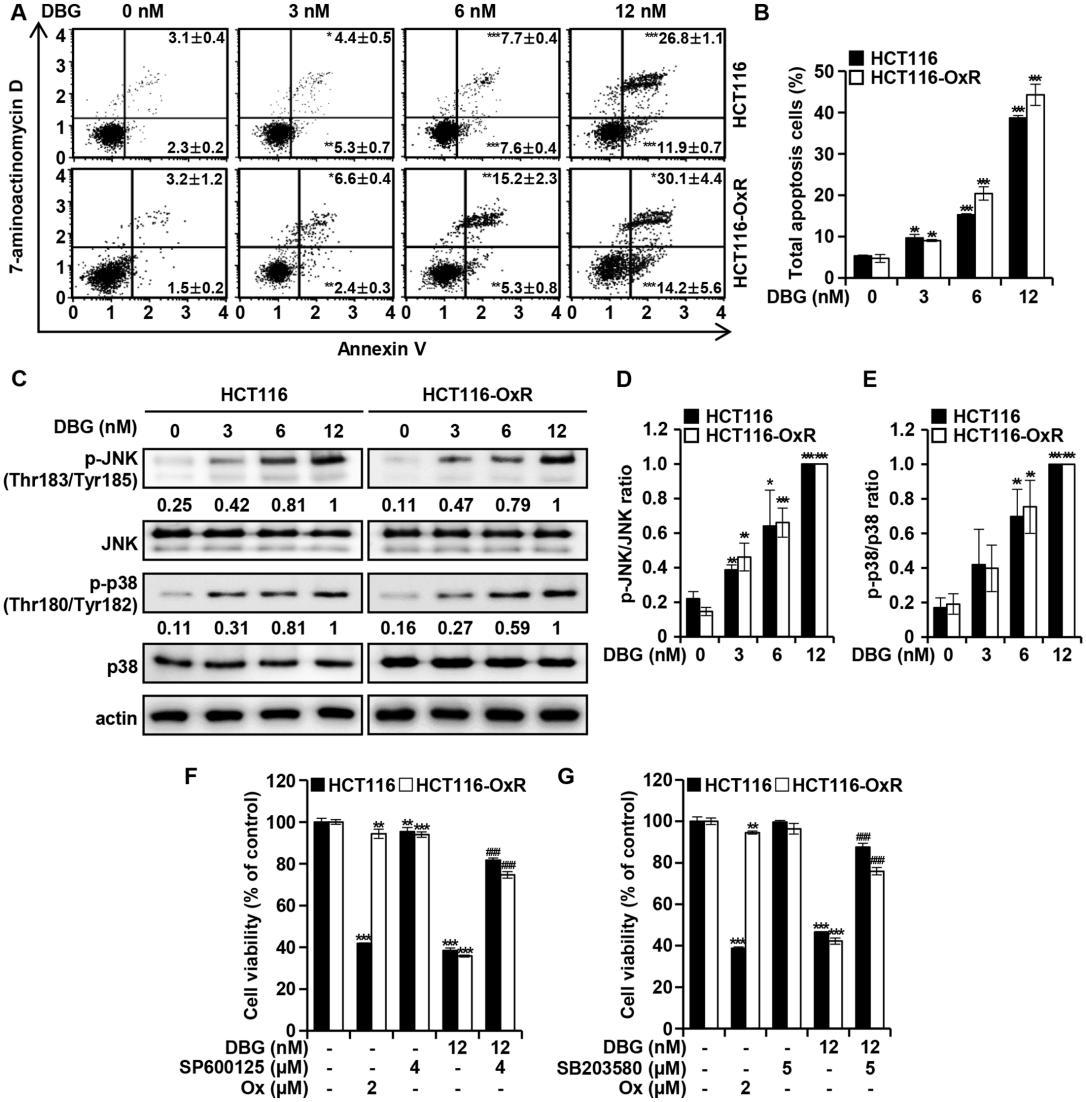
Deoxybouvardin glucoside (DBG)-induced apoptosis of colorectal cancer (CRC) cells mediated by JNK and p38 MAPK activation. HCT116 and HCT116-OxR cells were incubated with DBG (0, 3, 6, and 12 nM) for 48 h and subjected to flow cytometry analysis with Annexin V/7-AAD double staining. (**A**) Flow cytometry with Annexin V staining. (**B**) Ratio of apoptotic cells. *p < 0.05, **p < 0.01, and ***p < 0.001 compared to the control. (**C**) Western blot analysis of p-JNK, JNK, p-p38, and p38. Actin was used as a loading control. (**D-E**) Ratios of p-JNK/JNK and p-p38/p38. *p < 0.05, **p < 0.01, and ***p < 0.001 compared to the control. (F-G) Viability of CRC cells treated for 48 h with DBG, SP600125, SB203580, or Ox. **p < 0.01 and ***p < 0.001 compared to the control. ^###^*p* < 0.001 compared to DBG treatment.

To determine whether the anti-proliferative activity of DBG in CRC cells was mediated by the induction of apoptosis, flow cytometry was performed. The annexin V assay showed that DBG treatment increased the proportion of CRC cells undergoing apoptosis in a dose-dependent manner. The proportion of cells in the apoptotic phase induced by DBG treatment was greater than that in the untreated group at 48 h (Fig. 3A and 3B). In HCT116 cells, the ratio of cells in the apoptotic phase increased from the background level of 5.32 ± 0.22 to 9.65± 0.92, 15.26 ± 0.30, and 38.68 ± 0.61% after treatment with 3, 6, and 12 nM DBG, respectively. Similarly, the ratio of HCT116-OxR cells in the apoptotic phase increased from 4.73 ± 0.95 to 9.05 ± 0.24, 20.43 ± 1.61, and 44.28 ± 2.59%, respectively. To determine whether JNK and p38 MAPK activation was involved in DBG-induced apoptosis, the levels of related proteins were examined using western blot analysis (Fig. 3C). DBG increased the phosphorylation of JNK and p38 MAPK in a dose-dependent manner (Fig. 3D and 3E). To determine whether the activation of these kinases mediates DBG-induced apoptosis, we pretreated CRC cells with the JNK inhibitor SP600125 (4 μM) or the p38 MAPK inhibitor SB203580 (5 μM), and determined the viability of DBG-treated CRC cells (Fig. 3F and 3G). In HCT116 cells, the cell viability of the DBG only-treated group was 38.52 ± 1.08%, whereas that of the DBG and SP600125 pretreatment group was 81.76 ± 0.97%. Likewise, SP600125 reverted the cytotoxicity of DGB in HCT116-OxR cells: the viability of the DBG only-treated group was 35.80 ± 0.51% and that of the DBG and SP600125 pretreatment group was 74.63 ± 1.56%. Further, the viability of HCT116 and HCT116-OxR cells increased in the DBG-only treatment groups from 46.64 ± 0.09 and 42.25 ± 1.49% to 87.55 ± 1.85 and 75.94 ± 1.75%, respectively. These results suggest that activation of JNK and p38 MAPK mediates DBG-induced apoptosis.

### DBG Induces Cell Cycle Arrest in CRC Cells

**Fig. 4:**
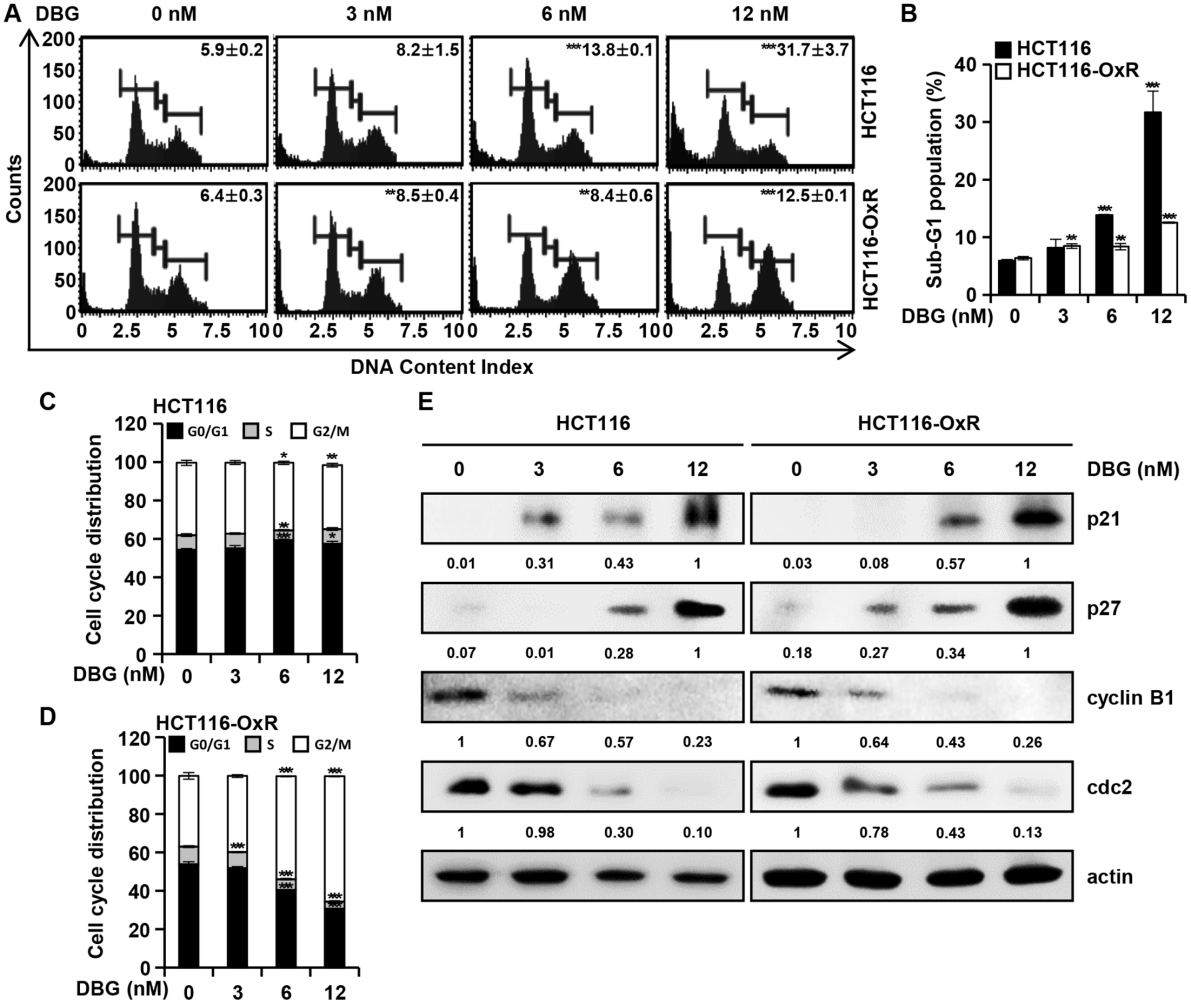
Cell cycle arrest induced by deoxybouvardin glucoside (DBG) in colorectal cancer (CRC) cells. CRC HCT116 and HCT116-OxR cells were incubated with DBG (0, 3, 6, and 12 nM) for 48 h. (**A**) Flow cytometry analysis with PI staining. (**B**) Ratio of cells in sub-G1 phase. **p < 0.01 and ***p < 0.001 compared to the control. (**C-D**) Cell cycle distribution. *p < 0.05, **p < 0.01, and ***p < 0.001 compared to the control. (**E**) Western blot analysis of p21, p27, cyclin B1, and cdc2. Actin was used as a loading control.

To examine the effect of DBG on cell cycle progression in CRC cells, flow cytometry was performed using the Muse^TM^ Cell Cycle Kit (Fig. 4A). The proportion of HCT116 cells in the sub-G1 phase increased from the background level of 5.90 ± 0.20 to 8.17 ± 1.45, 13.83 ± 0.12, and 31.70 ± 3.65% at 3, 6, and 12 nM DBG, respectively. In HCT116-OxR cells, the corresponding ratio increased from 6.37 ± 0.29 to 8.47 ± 0.40, 8.37 ± 0.55, and 12.53 ± 0.12%, respectively (Fig. 4B). In addition to the increase in the proportion of cells in the G1 phase, the proportion of HCT116 cells in the G2/M phase remained relatively unchanged (Fig. 4C, 37.73% in the control and 33.30% in the DBG 12 nM treatment). However, the ratio of G2/M-phase HCT116-OxR cells increased significantly with increasing DBG concentration: 36.80% in control and 65.40% in DBG 12 nM treatment (Fig. 4D). Western blotting was performed to examine the effect of DBG treatment on the levels of cell cycle-related proteins. DBG treatment resulted in the upregulation of p21 and p27 and downregulation of cyclin B1 and cdc2 (Fig. 4E).

### DBG Induces Apoptosis of CRC Cells through the Mitochondrial Pathway

**Fig. 5:**
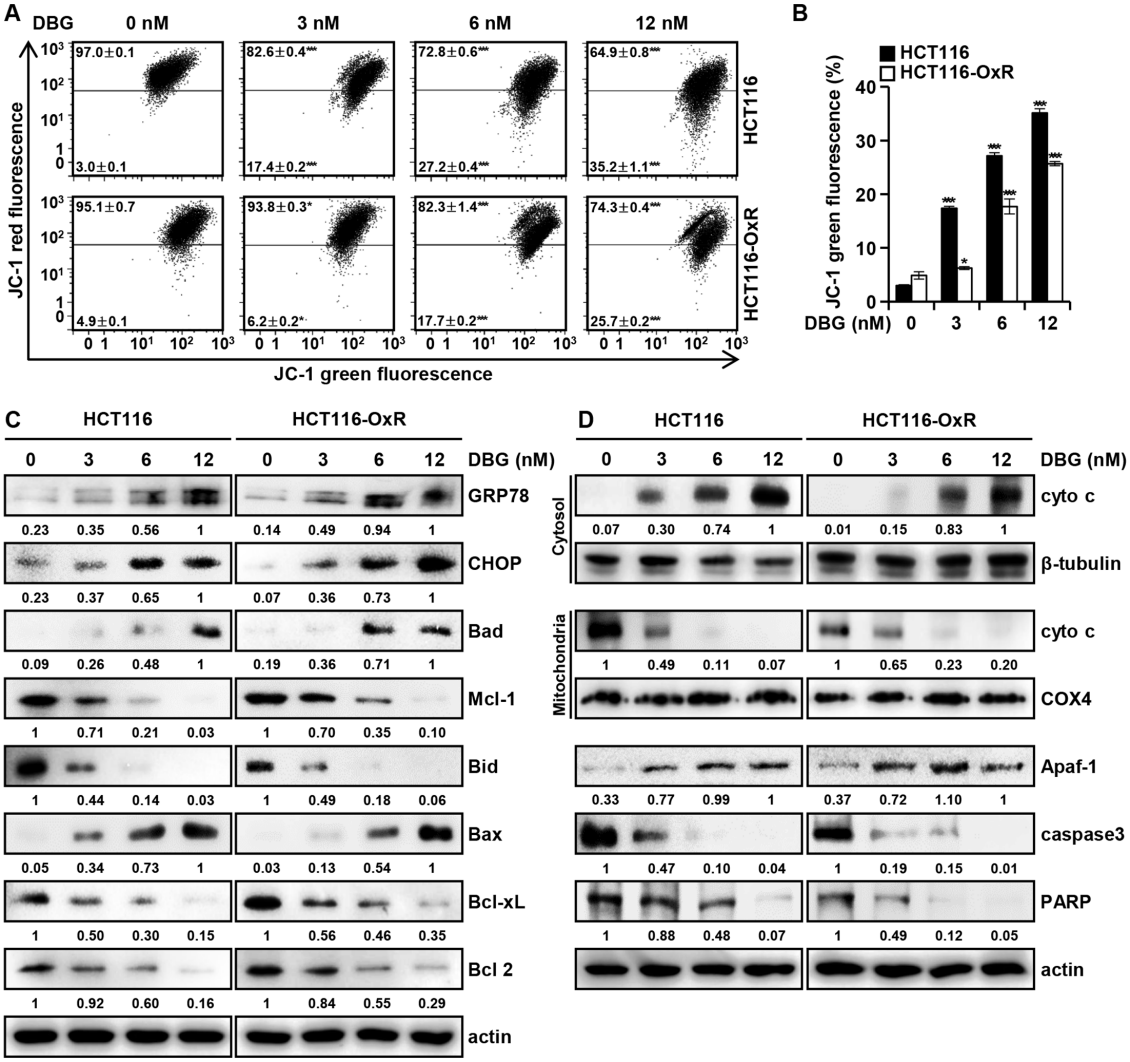
Mitochondrial membrane potential (MMP) disruption and permeabilization of mitochondrial membrane by deoxybouvardin glucoside (DBG) in colorectal cancer (CRC) cells. HCT116 and HCT116-OxR cells were incubated with DBG (0, 3, 6, and 12 nM) for 48 h. (**A**) Flow cytometric analysis with JC-1 staining. (**B**) Ratio of cells with depolarized mitochondrial membranes. *p < 0.05 and ***p < 0.001 compared to the control. (**C**) Western blot analysis of GRP78, CHOP, Bad, Mcl-1, Bid, Bax, Bcl-xL, and Bcl 2. (**D**) Western blot analysis of cytoplasmic and mitochondrial fractions of cytochrome c (cyto c), Apaf-1, caspase3, and PARP. β-tubulin was used for cytoplasmic fraction loading, COX4 for mitochondrial fraction loading, and actin was used as a total loading control.

To monitor the change in the mitochondrial membrane potential (MMP), flow cytometry analysis with JC-1 fluorescence staining was used (Fig. 5A). Treatment of CRC cells with DBG increased the ratio of JC-1 monomers/JC-1 aggregates, indicating mitochondrial membrane depolarization in accordance with DBG concentration (3, 6, and 12 nM). In HCT116 cells, the proportion of JC-1 monomers increased from the background 2.99 ± 0.12 to 17.37 ± 0.37, 27.17 ± 0.57, and 35.15 ± 0.79% by DBG treatment at 3, 6, and 12 nM, respectively. The corresponding values in HCT116-OxR cells increased from the background 4.86 ± 0.70% to 6.24 ± 0.28, 17.71 ± 1.37, and 25.68 ± 0.40%, respectively (Fig. 5B). MMP disruption may result in cytochrome c release from the mitochondria into the cytoplasm. A shift in the balance of Bcl-2 family proteins was observed in western blot analysis: an increase in Bad and Bax levels and a decrease in Bid, Bcl-2, Bcl-xL, and Mcl-1 levels (Fig. 5C). Furthermore, western blot analysis of the mitochondrial and cytoplasmic fractions indicated that DBG treatment decreased the level of mitochondrial cytochrome c and increased that of cytoplasmic cytochrome c (Fig. 5D). Additionally, an increase in the level of Apaf-1 and a decrease in the levels of full-length PARP and caspase3 were observed. Decrease in full-length caspase3 indicates an activation of caspase3 by proteolytic cleavage. These results suggested that DBG induces apoptosis in CRC cells through the mitochondrial pathway.

### DBG Induces the Activation of Caspases

**Fig. 6:**
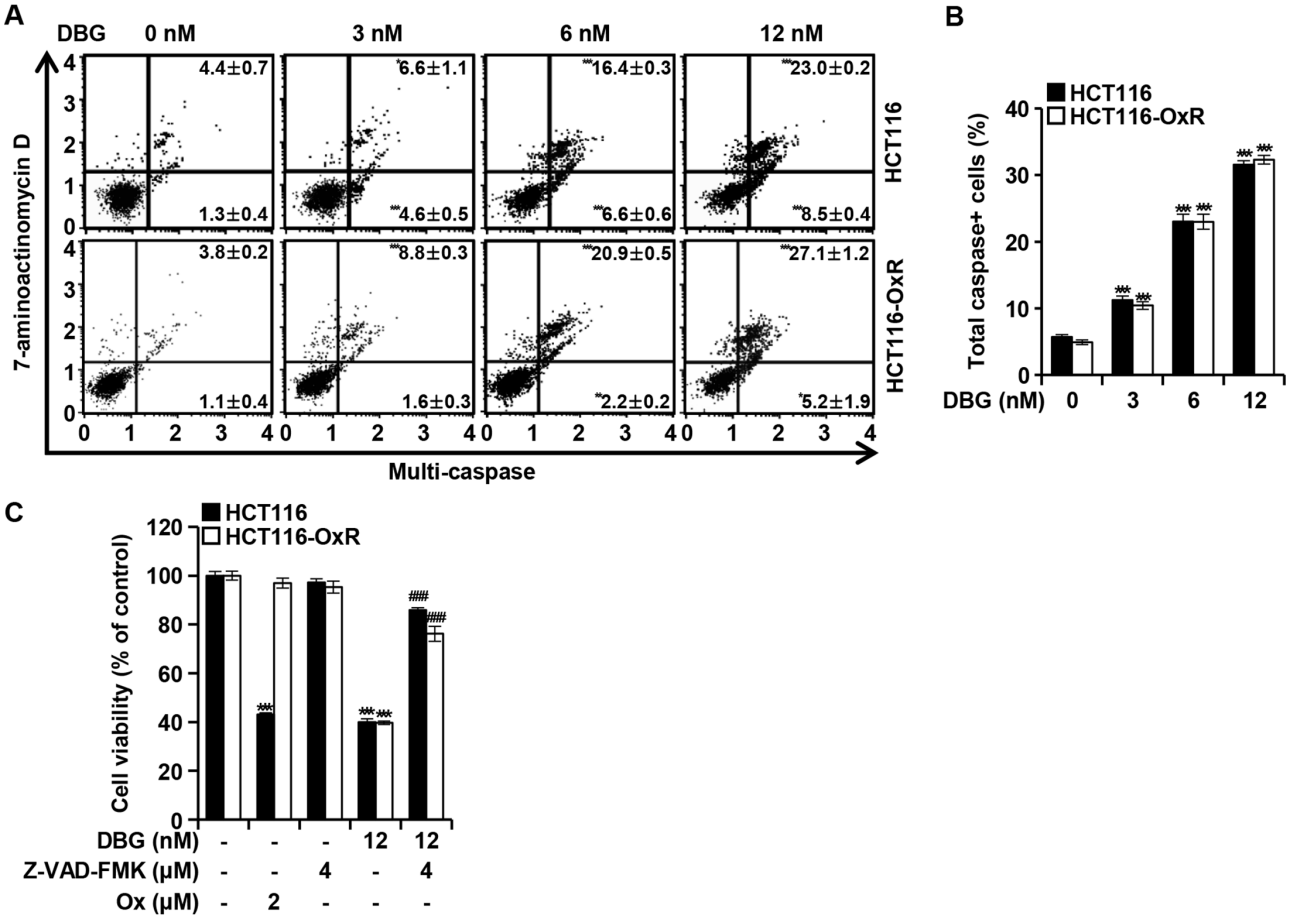
Caspase activation by deoxybouvardin glucoside (DBG) in colorectal cancer (CRC) cells. CRC HCT116 and HCT116-OxR cells were incubated with DBG (0, 3, 6, and 12 nM) for 48 h. (**A**) Flow cytometry analysis with Muse^TM^ MultiCaspase Kit. *p < 0.05, **p < 0.01, and ***p < 0.001 compared to the control. (**B**) Ratio of cells with activated caspases. ***p < 0.001 compared to the control. (**C**) Viability of CRC cells treated for 48 h with DBG, Z-VAD-FMK, and Ox. ***p < 0.001 compared to the control. ^###^*p* < 0.001 compared to DBG treatment.

To determine the activation of caspases induced by DBG treatment, flow cytometry was performed using the Muse^TM^ MultiCaspase Kit (Fig. 6A). An increase in the number of cells with activated caspases was observed in a DBG concentration-dependent manner (Fig. 6B). To determine whether the activation of caspases mediated DBG-induced apoptosis, we incubated CRC cells with DBG, Z-VAD-FMK, and Ox for 48 h. Notably, co-treatment with DBG and Z-VAD-FMK resulted in a cell viability equivalent to that in the control (Fig. 6C). Z-VAD-FMK (4 μM) alone did not affect cell viability, and DBG-induced apoptosis was reduced in both HCT116 and HCT116-OxR cells. These results illustrate that DBG-induced apoptosis in CRC cells is mediated by caspase activation.

### DBG Induces ROS Production to Induce CRC Cell Apoptosis

**Fig. 7:**
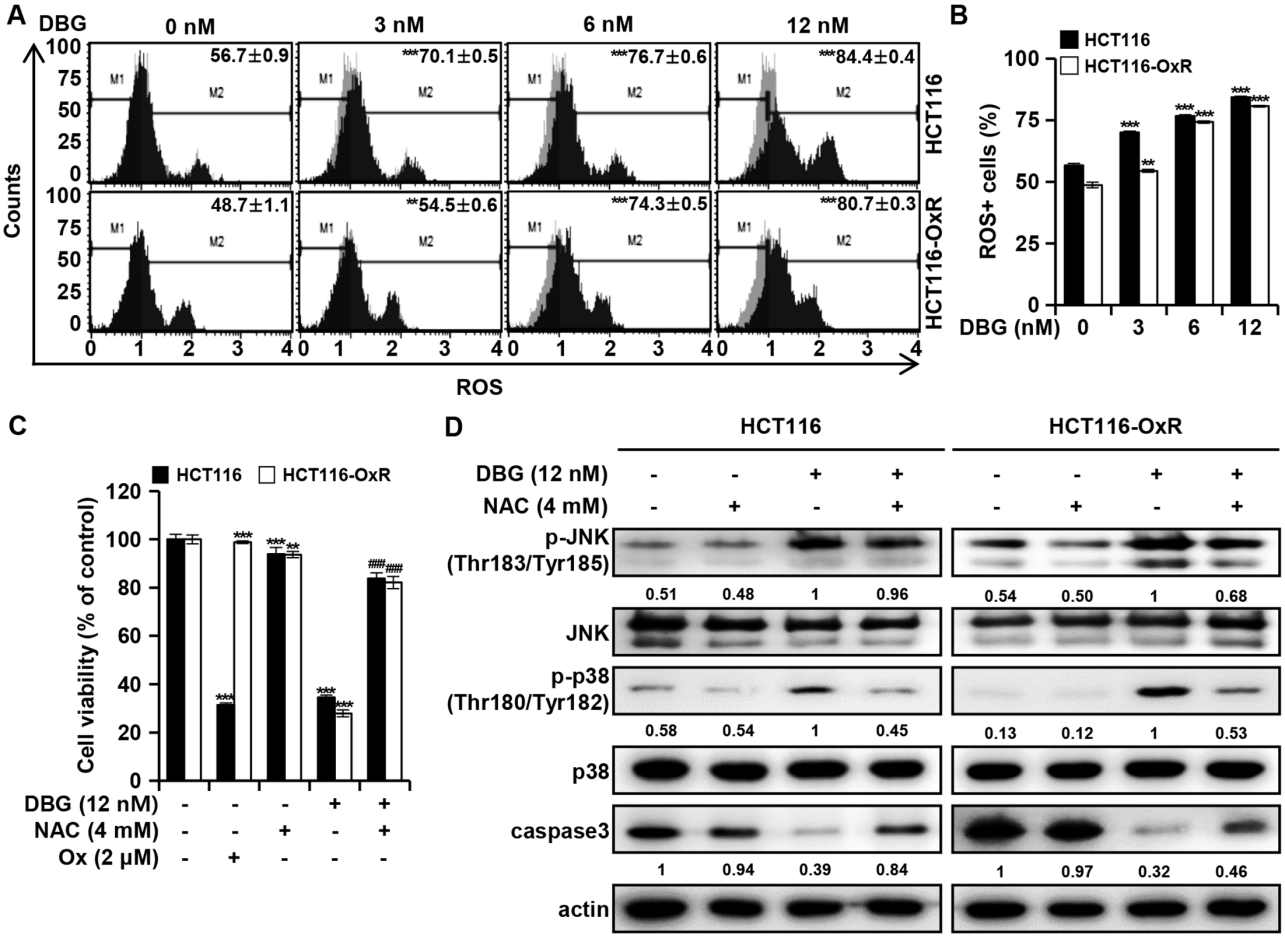
Reactive oxygen species (ROS) elevation by deoxybouvardin glucoside (DBG) in colorectal cancer (CRC) cells. CRC HCT116 and HCT116-OxR cells were incubated with DBG (0, 3, 6, and 12 nM) for 48 h. (**A**) Flow cytometry with Muse^TM^ Oxidative Stress Kit and ratio of cells with high ROS levels. (**B**) Ratio of cells with ROS **p < 0.01 and ***p < 0.001 compared to the control. (**C-D**) CRC cells were incubated with DBG, NAC, or Ox, as indicated. (**C**) Cell viability analysis **p < 0.01 and ***p < 0.001 compared to the control. ^###^*p* < 0.001 compared to DBG treatment. (**D**) Western blot analysis of p-JNK, JNK, p-p38, p38, and caspase3 levels. Actin was used as a loading control.

Increased cellular ROS levels may induce apoptosis in cancer cells. Cellular ROS levels were determined using the Muse^TM^ Oxidative Stress Kit (Fig. 7A). The cellular ROS level of HCT116 cells increased from 56.65 ± 0.89% to 70.06 ± 0.53%, 76.72 ± 0.61%, and 84.41 ± 0.39% at 3, 6, and 12 nM DBG, respectively (Fig. 7B). The corresponding values in HCT116-OxR cells increased from 48.72 ± 1.13 to 54.49 ± 0.58, 74.30 ± 0.51, and 80.70 ± 0.34%, respectively. These results indicated that DBG treatment upregulated ROS levels in CRC cells. To investigate the role of increased ROS levels, we examined the viability of CRC cells treated with DBG 12 nM for 48 h with or without pretreatment with the ROS scavenger NAC (4 mM) for 3 h. Pretreatment of CRC cells with NAC significantly prevented DBG-induced apoptosis (Fig. 7C). Moreover, the increase in p-JNK and p-p38 levels and decrease in caspase-3 levels induced by DBG treatment were reversed by NAC pretreatment (Fig. 7D). These results implied that the accumulation of ROS mediated DBG-induced apoptosis was a result of upstream JNK and p38 signaling.

## Discussion

The anti-inflammatory and anti-cancer activities of DB have been reported in several model systems [4, 7]. We hypothesized that DBG, a glucoside derivative of DB, in which glucose is linked to a cyclic hexapeptide, could exert antiproliferative activity in CRC cells. We demonstrated that DBG induced apoptosis in CRC cells to inhibit their growth. We observed that DBG was antiproliferative in both Ox-sensitive and Ox-resistant CRC HCT116 cells but not in HEKa cells. While the anticancer activity of DBG would be more apparent if a broader range of CRC cell lines and non-cancerous colon epithelial cells are tested, our results suggest that DBG is more likely cancer-selective (Fig. 2). Ox-resistance is involved with several mechanisms: defects in apoptosis, drug efflux, impaired DNA adduct formation, and so on [15]. HCT116-OxR cells release growth factors like progranulin as one of resistance mechanisms [17]. It is intriguing to consider whether DBG inhibits the secretion of growth factors in HCT116-OxR cells, warranting further investigation. Initially, DBG demonstrated effective antiproliferative activity in HCT116-OxR cells, suggesting that DBG itself or its molecular targets could provide valuable insights into overcoming Ox resistance in chemotherapy.

Apoptosis, or programmed cell death, is an orchestrated cell death process that can be initiated by diverse stimuli, such as cell stress, DNA damage, and immune reactions [18]. Apoptosis induction is one strategy for treating cancer cells with promising results. Several chemotherapeutic agents such as 5-fluorouracil [19] and oxaliplatin [20] exert anticancer effects by inducing apoptosis. Annexin V staining indicated that DBG induced apoptosis in CRC cells (Fig. 3). The MAPK signaling pathway is involved in diverse biological processes through several cellular mechanisms. Early studies indicate that MAPK plays an important role in turning cellular stimuli into cellular responses, including growth, differentiation, migration, proliferation, and apoptosis [21]. JNK and p38 MAPK are usually upregulated if activated upon stimulation by physical, chemical, or biological stresses to the cell, whereas the ERK1/2 signaling mainly responds to cellular growth factors [22]. JNK and p38 MAPK activation plays an important role in maintaining the balance between cell survival and death in response to cellular and other stresses [23]. We demonstrated that DBG induced the phosphorylation of JNK and p38 MAPK in HCT116 and HCT116-OxR cells, implying that DBG induced activation of the MAPK signaling pathway. Cell cycle progression can be blocked by various factors, and cell cycle regulation is one of the main targets of anticancer drugs [25]. Ox has been reported to accumulate in cells in the G2/M phase [26], and our data indicated that DBG partially increased the ratio of CRC cells in the G1 phase (Fig. 4A and 4B). An increase in G2/M-phase cell numbers was also noted in HCT116-OxR cells, but not in HCT116 cells (Fig. 4C and 4D). G2/M phase arrest and an increase in the levels of p21 and p27 are often related [27], which function as inhibitors of cyclin B1/cdc2 [28]. Notably, majority of the cells were in sub-G1 phase undergoing cell death suggesting that induction of apoptosis is more relevant in the antitumor activity of DBG.

Depolarization of the mitochondrial membrane is a key event in apoptosis progression [29], resulting from an imbalance in the Bcl-2 family of proteins [30]. DBG treatment resulted in the depolarization of CRC cells (Fig. 5A and 5B). Further, a shift in the balance between the pro- and anti-apoptotic Bcl-2 family proteins was observed [31] (Fig. 5C). This led to the permeabilization of mitochondrial membranes and release of Cytochrome C from the mitochondria into the cytoplasm, followed by elevation of Apaf-1 protein levels and execution of apoptosis characterized by the cleavage of PARP and pro-caspase3 (Fig. 5D).

Caspase activation is a hallmark of apoptosis [32]. The activation of caspases, assessed using the Muse^TM^ MultiCaspase Assay Kit, was increased by DBG treatment in a concentration-dependent manner (Fig. 6A and 6B), signifying the role of caspase activation in DBG-induced apoptosis. The prevention of DBG-induced apoptosis by pretreatment with Z-VAD-FMK (Fig. 6C) indicated that DBG-induced apoptosis was mediated by caspase activation.

ROS are involved in diverse physiological and pathophysiological cellular functions [33]. The ROS level in cancer cells is considered to be slightly higher than that in non-cancer cells, and is considered beneficial for cancer cell survival [34]. However, the generation of ROS levels beyond the capacity of cancer cells may result in cytotoxicity and apoptosis [35]. DBG treatment elevated cellular ROS levels in CRC cells (Fig. 7A and 7B). Moreover, prevention of DBG-induced apoptosis by pretreatment with NAC 4 mM (Fig. 7C) indicated that excessive ROS generation mediated apoptotic progression. Notably, phosphorylation of the protein kinases JNK and p38 MAPK by DBG treatment was reversed by NAC to some extent, illustrating that ROS generation is an upstream signaling pathway of apoptosis (Fig. 7D).

It could be argued whether this study holds significance, given the existence of numerous natural products demonstrating similar anticancer activity by reducing ROS. First, the anticancer effect of DBG is involved with the induction of apoptosis through ROS generation and inhibition of JNK and p38 MAPK, not the reduction of ROS. It should be noted that numerous natural products employ a variety of mechanisms to exert anticancer effects, and it cannot be collectively attributed to antioxidant effects of natural products [36]. For example, triptonide inhibits the growth of CRC cells by inducing ferroptosis—a type of cell death driven by iron—rather than apoptosis [37]. Second, DBG exhibits a notably potent anticancer effect, with an IC_50_ in the nanomolar range, which is more effective than many other phytochemicals. While honokiol, a phenolic compound derived from Magnolia, promotes apoptosis in CRC cells by upregulating p53, the antiproliferative potency of honokiol is nearly a thousand times weaker than that of DBG [38]. Third, DBG is a cyclic peptide glycoside, unlike other small molecule phytochemicals. Peptide drugs usually have low toxicity profiles [39], and there exist several structural benefits in cyclic peptides such as better specificity, possibility of crossing the cell membrane [40]. Although the key event triggering ROS induction following DBG treatment has yet to be determined, DBG stands out as particularly unique compared to other phytochemicals in many aspects.

Our recent study regarding the antiproliferative activity of DB in CRC cells [9] allowed us to compare DB and DBG. It was observed that IC_50_ value for DBG was a little bit higher than that of DB. Typically, glycosides show enhanced water solubility [41]. We believe that both DB and DBG exert their antitumor activity within cells. DBG, a glycoside form, would have an advantage in water solubility even though IC_50_ is higher, and further research could help identify their molecular targets. It remains to be seen whether DB and DBG enter the cell, possibly via a glucose transporter, or by directly passing through the membrane with their unique cyclic peptide structure.

In conclusion, we demonstrated for the first time that DBG exhibits anticancer activity against both Ox-sensitive and Ox-resistant CRC HCT116 and HCT116-OxR cells. DBG induced cell cycle arrest at the G2/M phase, caused mitochondrial membrane depolarization, and activated caspases to trigger apoptosis in CRC cells through ROS-mediated activation of JNK and p38 MAPK. Further studies in animal models would validate the potential of DBG in treating CRC, regardless of Ox-resistance, and to deepen our understanding of its anticancer activity.

## Supplemental Materials

Supplementary data for this paper are available on-line only at http://jmb.or.kr.



## Figures and Tables

**Table 1:** ^1^H NMR and ^13^C NMR spectroscopy data of deoxybouvardin glucoside (in CD_3_OD, 300 and 75 MHz).

Position		^1^H	^13^C
Ala1	α	4.48 (m)	48.1
	β	1.26 (ov)	21.0
	C=O		173.4
Ala2	α	4.76 (ov)	45.6
	β	1.33 (ov)	16.4
	C=O		174.6
Tyr3	α	3.73 (ov)	68.3
	β	3.33 (m)	33.7
	γ		132.3
	δ*2	7.10 (ov)	131.9
	ε*2	6.68 (d, *J* = 8.1 Hz)	115.0
	ζ		160.0
	C=O		171.0
	NMe	2.94 (s)	40.3
	OMe	3.80 (s)	55.7
Ala4	α	4.76 (ov)	47.7
	β	1.12 (d, *J* = 6.6 Hz)	18.8
	C=O		173.0
Tyr5	α	5.48 (dd, *J* = 11.4, 9.3 Hz)	55.7
	βa	2.70 (d, *J* = 11.4 Hz)	37.4
	βb	3.60 (ov)	
	γ		137.0
	δa	7.18 (ov)	134.1
	δb	7.53 (ov)	131.9
	εa	7.33 (ov)	127.3
	εb	6.83 (m)	125.1
	ζ		159.8
	C=O		171.3
	NMe	3.09 (s)	31.1
Tyr6	α	4.65 (ov)	58.7
	βa	3.17 (ov)	36.6
	βb	3.48 (ov)	
	γ		131.5
	δa	6.84 (dd, *J* = 8.6, 2.6 Hz)	122.6
	δb	3.80 (s)	115.8
	εa	7.10 (ov)	118.7
	εb		154.7
	ζ		145.4
	C=O		172.2
	NMe	2.65 (s)	30.1
Glucose	1'	5.02 (d, *J* = 7.7 Hz)	102.9
	2'	3.57 (m)	74.9
	3'	3.51 (m)	77.9
	4'	3.45 (m)	71.4
	5'	3.79 (ov)	78.2
	6'	3.89 (ov)	62.5
