# Supplementary figures and images for: Deoxybouvardin Glucoside Induces Apoptosis in Oxaliplatin-Sensitive and -Resistant Colorectal Cancer Cells via Reactive Oxygen Species-Mediated Activation of JNK and p38 MAPK
Source: J Microbiol Biotechnol. 2025 Jan 30;35:e2410008. doi: 10.4014/jmb.2410.10008 (PMC11876011; doi:10.4014/jmb.2410.10008)

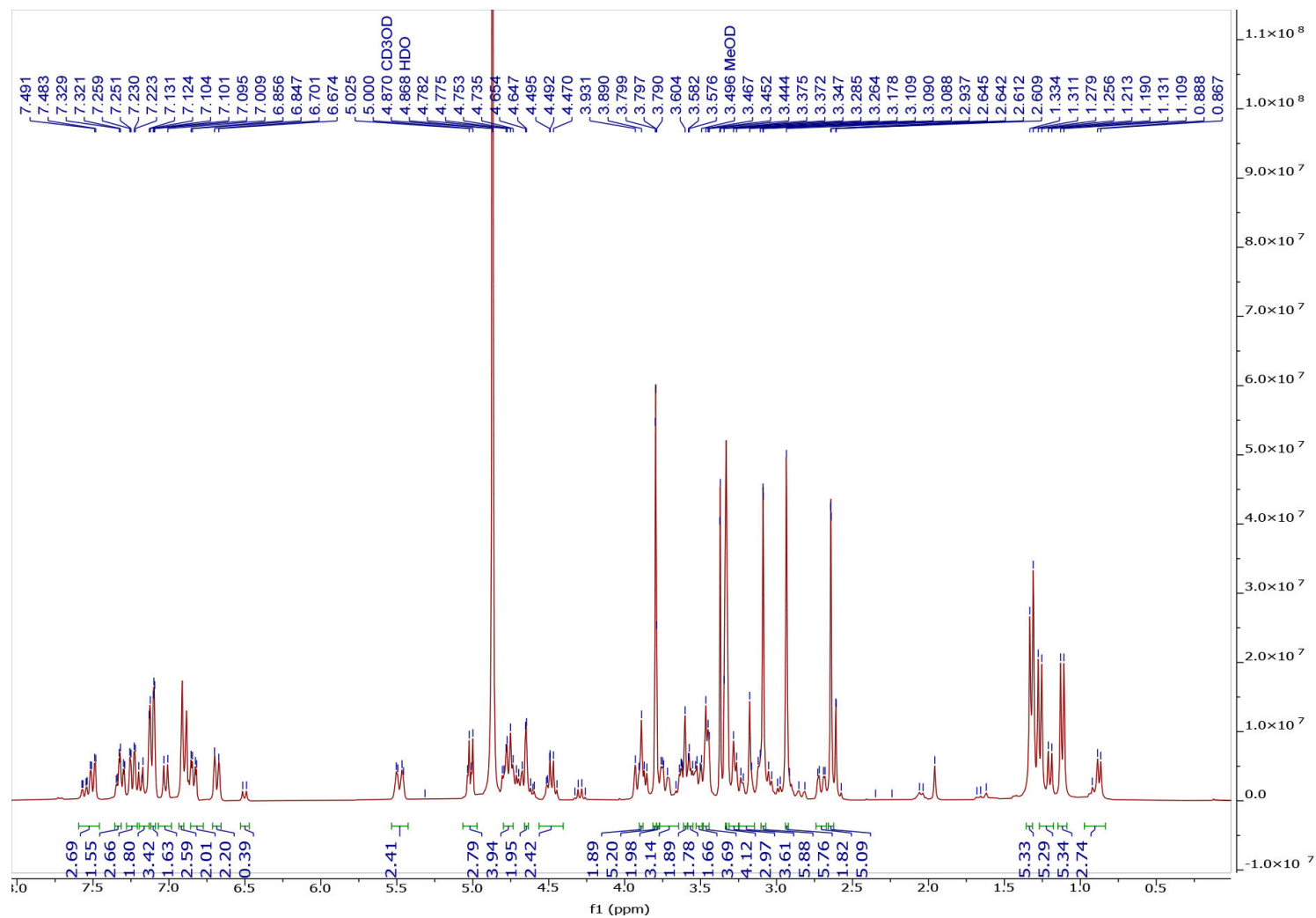

Figure S1.tif

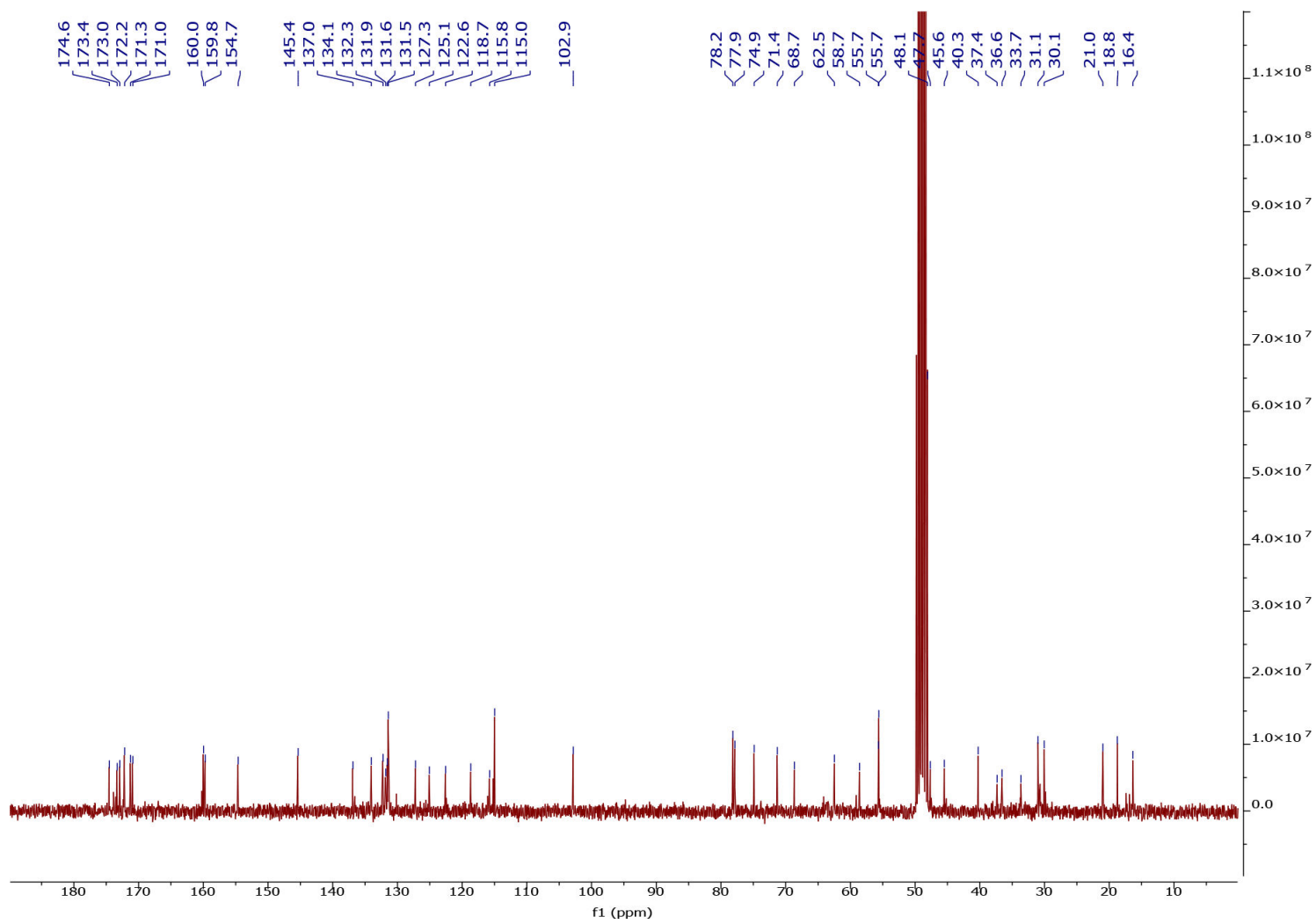

Figure S2.tif

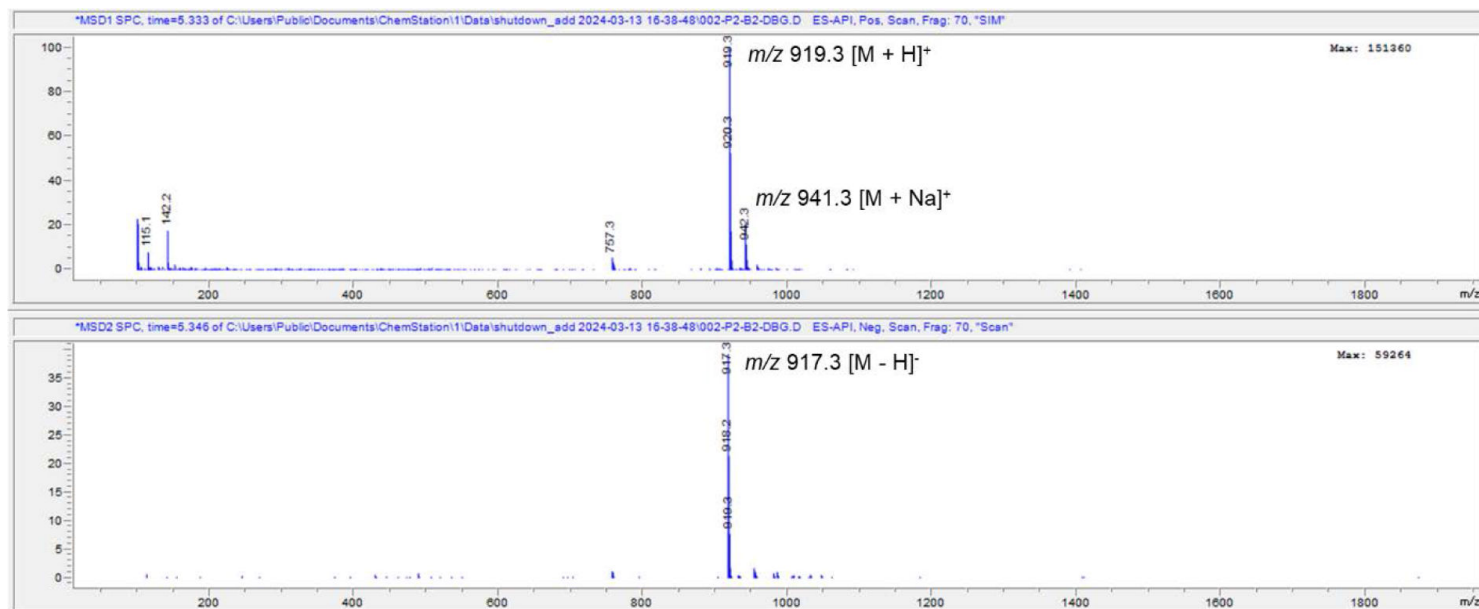

Figure S3.tif

Supplement: Supplementary file 1 [file jmb-35-e2410008-supple.pdf]
